# Crystal structure, Hirshfeld surface and crystal void analysis of (1*Z*)-1-[(*E*)-2-(2*H*-1,3-benzodioxol-5-yl­methyl­idene)hydrazin-1-yl­idene]-1,2-di­hydro­phthalazine

**DOI:** 10.1107/S2056989026000538

**Published:** 2026-01-23

**Authors:** Meiyazhagan Manvizhi, Srinivasan Senthilkumar, Sivashanmugam Selvanayagam

**Affiliations:** a Department of Chemistry Annamalai University, Annamalainagar Chidambaram 608 002 India; b PG & Research Department of Physics, Government Arts College, Melur 625 106, India; Vienna University of Technology, Austria

**Keywords:** hydrazone, inter­molecular hy­dro­gen bonds, Hirshfeld surface analysis, crystal structure

## Abstract

The title com­pound, C_16_H_12_N_4_O_2_, crystallizes in the tetra­gonal space group *I*4_1_/*a* with one mol­ecule in the asymmetric unit. The 2*H*-1,3-benzodioxole ring is almost planar [maximum deviation of −0.039 (3) Å] and makes a dihedral angle of 11.25 (5)° with the 1,2-di­hydro­phthalazine moiety. An intra­molecular N—H⋯N hy­dro­gen bond stabilizes the mol­ecular conformation. Inter­molecular N—H⋯N hy­dro­gen bonds, as well as C—H⋯N hy­dro­gen bonds, lead to the formation of supra­molecular chains extending parallel to [001]. The inter­molecular inter­actions were qu­anti­fied and analysed using Hirshfeld surface analysis, revealing that H⋯H inter­actions contribute most to the crystal packing (37.4%). The volume of the crystal voids and the percentage of free space were calculated to be 614.55 Å^3^ and 11.5%, respectively.

## Abstract 2

The main supra­molecular motif in the packing of the title com­pound is the formation of N—H⋯N and C—H⋯N hy­dro­gen-bonded chains extending parallel to [001].

## 1. Chemical context

Hydrazone derivatives have remained important mol­ecules in medicinal and organic chemistry for decades, largely because they are easy to synthesize, adaptable in structure and capable of exhibiting a wide range of biological activities. Within this class, hydrazones linked to heterocyclic systems have received particular attention. The presence of fused aromatic rings often strengthens pharmacological effects, and many such derivatives have been reported to show anti­diabetic, anti­cancer, anti­microbial, anti­oxidant and anti-inflammatory properties. The hydrazone unit (–C=N—NH–; Punitha *et al.*, 2020 ▸; Senthilkumar *et al.*, 2021 ▸) provides a conjugated path­way that allows for efficient electron delocalization and sup­ports inter­molecular hy­dro­gen bonding, both of which can improve inter­actions with biological receptors. The 1,2-di­hy­dro­iso­quinoline core is a widely recognized pharmacophore and often contributes to mol­ecular rigidity, planarity and the possibility of π–π stacking, characteristics associated with notable anti­cancer and neuroactive potential. The benzo[*d*][1,3]dioxole ring is a structural motif commonly encountered in naturally occurring bioactive com­pounds, such as safrole derivatives and piperine analogues. Its electron-rich O atoms, com­pact ring system and moderate lipophilicity help to enhance membrane permeability and to enable additional hy­dro­gen-bonding or π-based inter­actions. The combined influence of the electron-donating dioxole group and the electron-withdrawing hydrazone segment generates an inter­nal charge-transfer environment, a feature often linked with stronger biological responses, improved anti­oxidant behaviour and distinctive electronic transitions. Overall, the thoughtful assembly of these functional groups results in a mol­ecular scaf­fold with promising physicochemical and biological attributes (Maheswari *et al.*, 2025 ▸; Senthilkumar *et al.*, 2020 ▸).
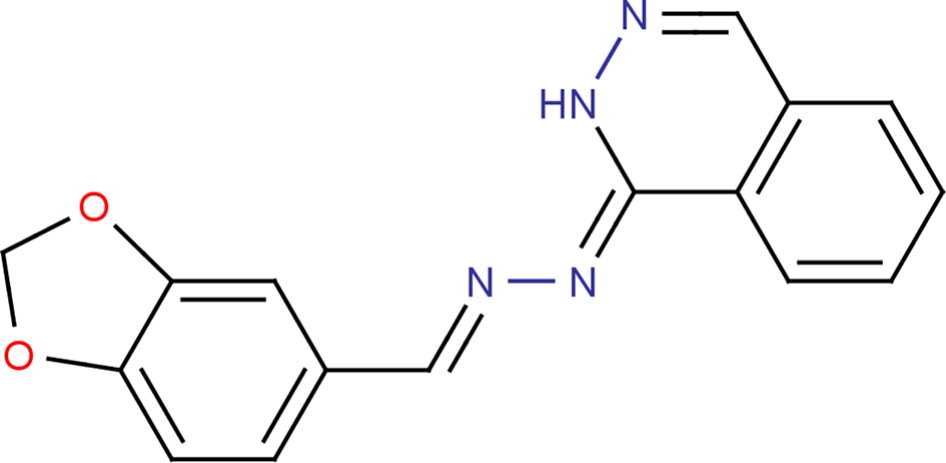


In the present work, we report on the crystal structure, Hirshfeld surface analysis and crystal void studies of (1*Z*)-1-[(*E*)-2-(2*H*-1,3-benzodioxol-5-yl­methyl­idene)hydrazin-1-yl­idene]-1,2-di­hydro­phthalazine, (I), which brings together several structural features discussed above.

## 2. Structural commentary

**Figure 1:**
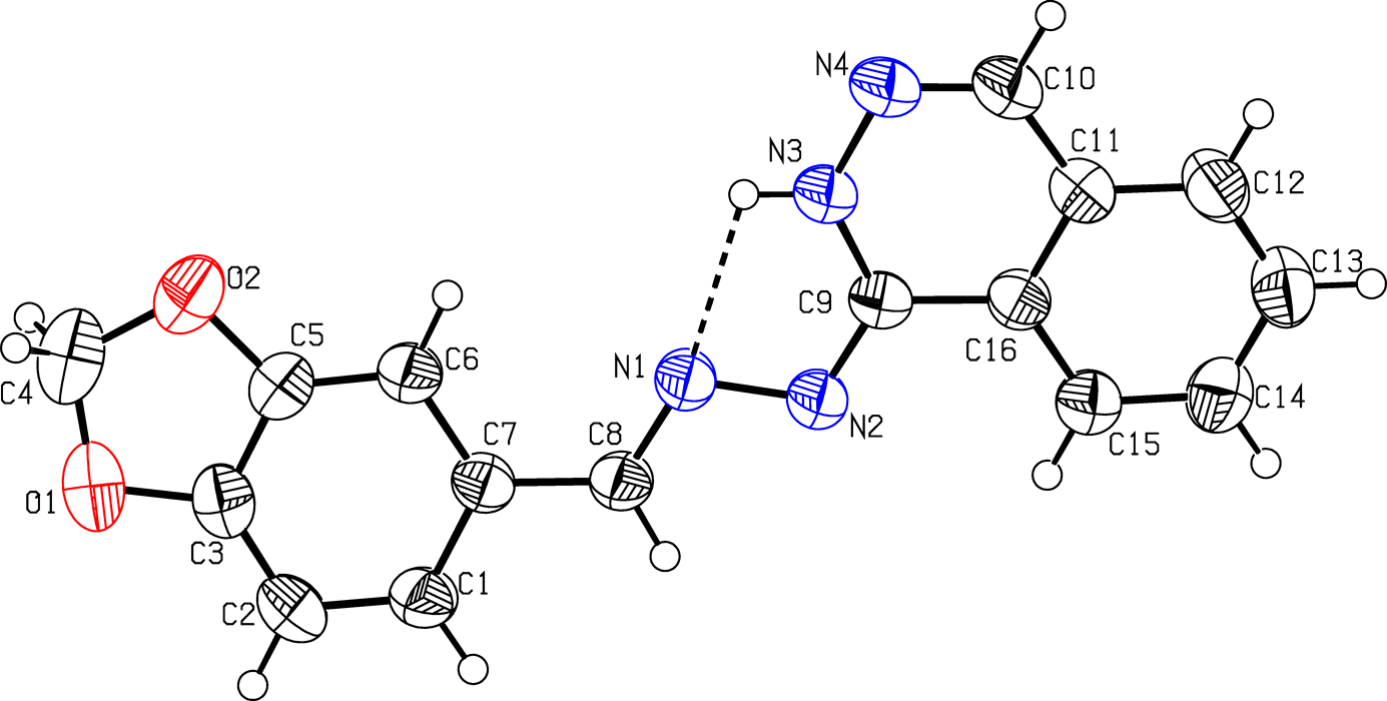
The mol­ecular structure of com­pound (I), showing the atom labelling. Displacement ellipsoids are drawn at the 50% probability level. The intra­molecular hy­dro­gen bond is shown as a dashed line.

The mol­ecular structure of (I) is displayed in Fig. 1 ▸. The C8—N1 [1.282 (2) Å], C9—N2 [1.308 (2) Å] and C10—N4 [1.289 (3) Å] bond lengths confirm their double-bond character. The 2*H*-1,3-benzodioxole ring (C1–C3/O1/C4/O2/C5–C7) is essentialy planar, with a maximum deviation of −0.039 (3) Å for atom C4. The planes of the fused five- and six-membered rings of this moiety make a dihedral angle of 0.41 (1)°. The 1,2-di­hydro­phthalazine moiety (C9/N3/N4/C10–C16) is also planar, exhibiting a maximum devation of 0.006 (2) Å for atom C15. The planes of the two fused six-membered rings of this moiety make a dihedral angle of 0.31 (1)°. The benzodioxole and di­hydro­phthalazine moieties are oriented with respect to each other at a dihedral angle of 11.25 (5)°. An intra­molecular N3—H3⋯N1 hy­dro­gen bond contributes to the stabilization of the mol­ecular conformation (Table 1 ▸) and generates an *S*(5) ring motif (Bernstein *et al.*, 1995 ▸), as shown in Fig. 1 ▸.

## 3. Supra­molecular features

**Figure 2:**
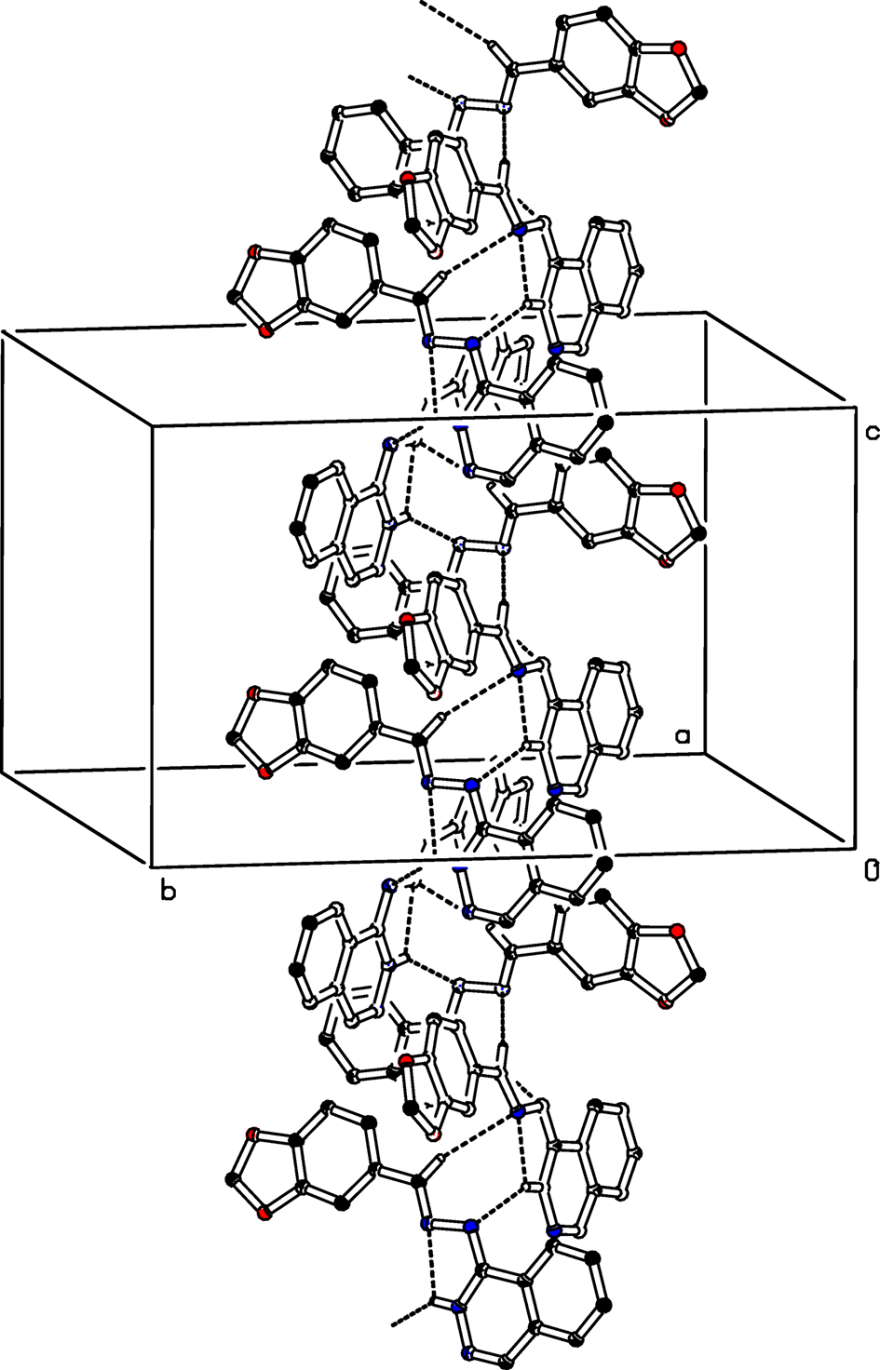
The crystal packing of (I), with N—H⋯N and C—H⋯N inter­molecular inter­actions shown as dashed lines. For clarity, H atoms not involved in these inter­actions have been omitted.

In the crystal structure of (I), the mol­ecules are inter­con­nected through N3—H3⋯N2^i^ hy­dro­gen bonds, generating a *C*(4) chain motif that propagates parallel to [001] (Table 1 ▸ and Fig. 2 ▸). In addition, non-classical C8—H8⋯N1^ii^ hy­dro­gen bonds link adjacent mol­ecules to form *C*(3) chain motifs that extend in a helical fashion parallel to [001]. Together with the intra­molecular N3—H3⋯N2 hy­dro­gen bond, these inter­actions combine into an *S*(7) ring motif, which further reinforces the overall crystal packing, as illustrated in Fig. 2 ▸.

## 4. Hirshfeld surface and void analysis

**Figure 3:**
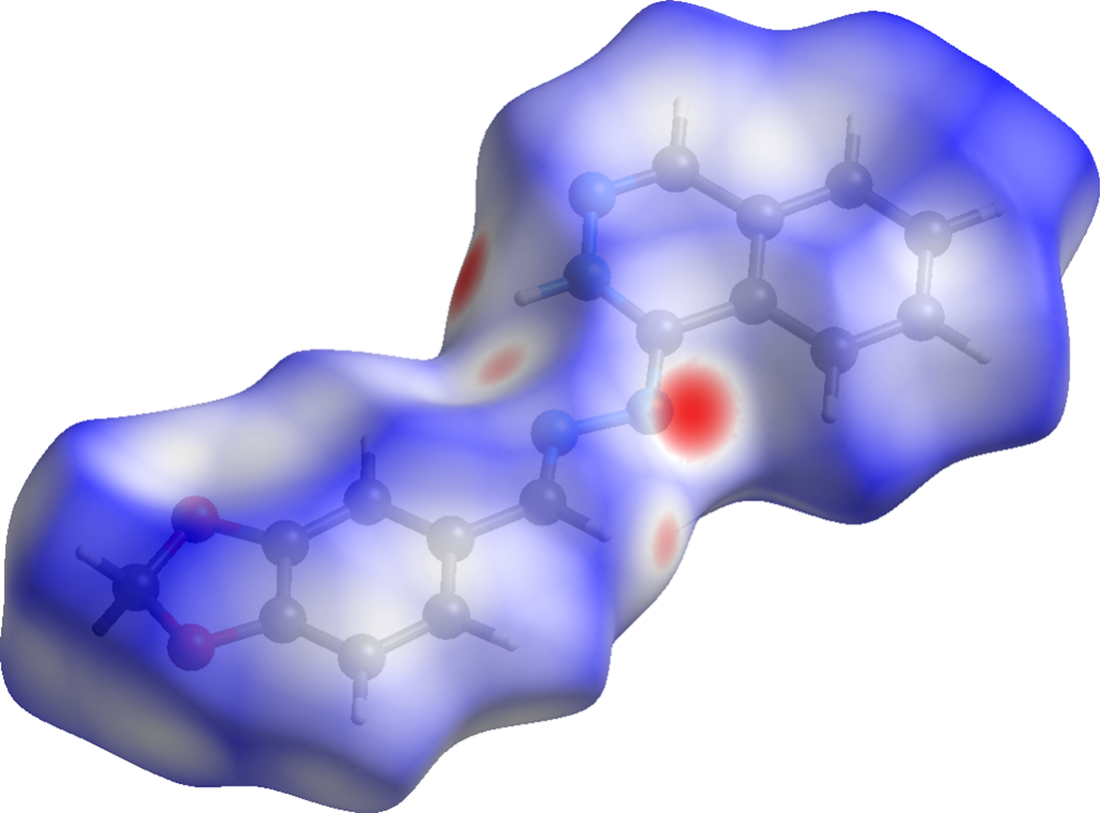
A view of the Hirshfeld surface mapped over *d*_norm_ for com­pound (I).

**Figure 4:**
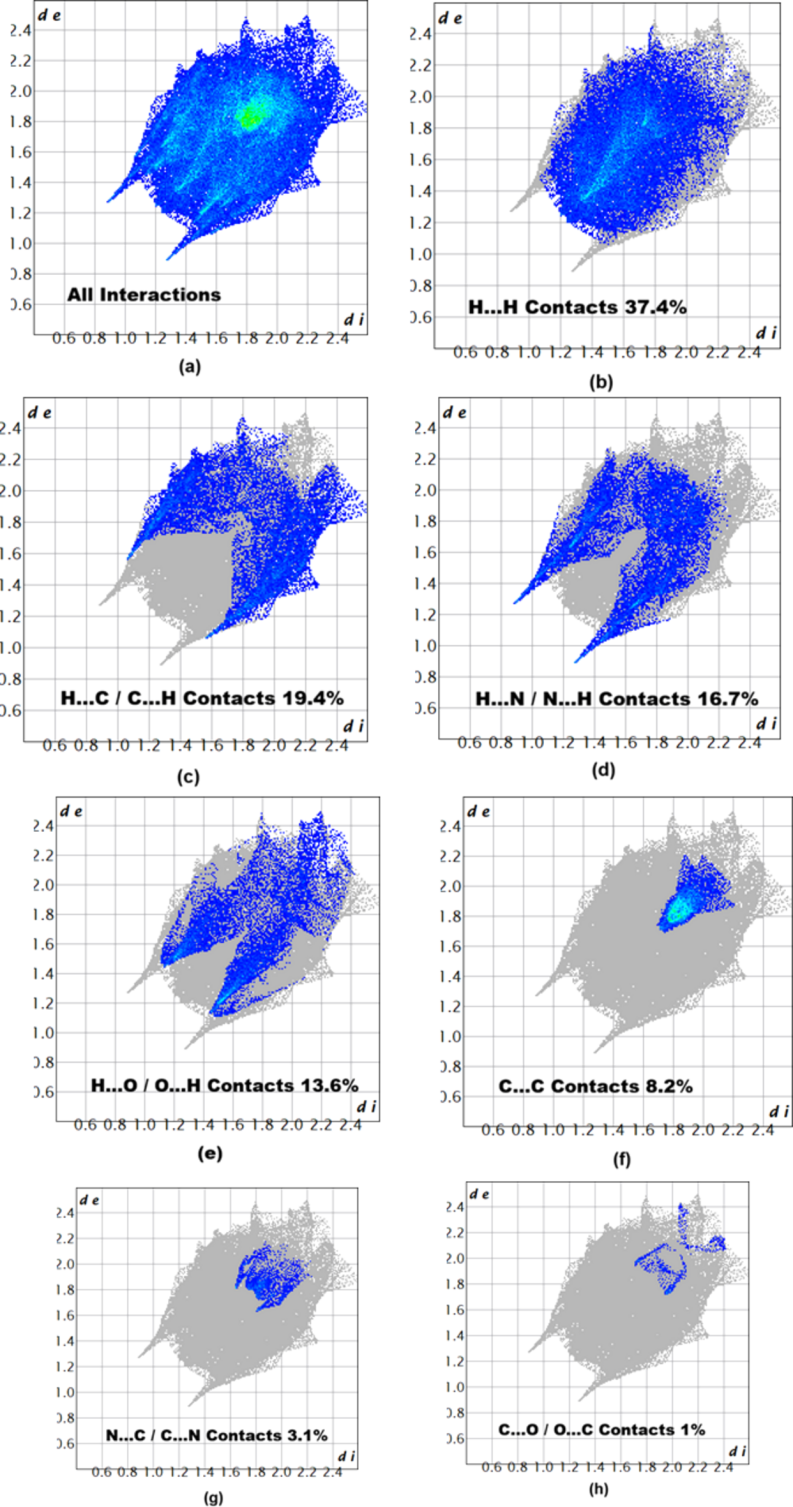
Two-dimensional fingerprint plots for com­pound (I), showing (*a*) all inter­actions, and delineated into (*b*) H⋯H, (*c*) H⋯C/C⋯H, (*d*) H⋯N/N⋯H, (*e*) H⋯O/O⋯H, (*f*) C⋯C, (*g*) N⋯C/C⋯N and (*h*) C⋯O/O⋯C inter­actions. The *d*_i_ and *d*_e_ values are the closest inter­nal and external distances (in Å) from given points on the Hirshfeld surface.

In order to characterize and qu­antify the inter­molecular in­ter­actions of (I), a Hirshfeld surface (HS) analysis (Spackman & Jayatilaka, 2009 ▸) was carried out using *CrystalExplorer* (Spackman *et al.*, 2021 ▸). The HS mapped over *d*_norm_ is illustrated in Fig. 3 ▸, where deep-red spots indicative of strong inter­actions occur at N1, N2, H3 and H8, and these atoms are responsible for the intra- and inter­molecular hy­dro­gen bonds discussed above. The associated two-dimensional fingerprint plots (McKinnon *et al.*, 2007 ▸) provide qu­anti­tative information about the non-covalent inter­actions in the crystal packing in terms of the percentage contribution of the inter­atomic contacts (Spackman & McKinnon, 2002 ▸). As shown in Fig. 4 ▸, the overall two-dimensional fingerprint plot for com­pound (I) is delineated into H⋯H, H⋯C/C⋯H, H⋯N/N⋯H, H⋯O/O⋯H, C⋯C, N⋯C/C⋯N and C⋯O/O⋯C contacts, re­vealing that H⋯H and H⋯C/C⋯H inter­actions are the main contributors to the crystal packing.

**Figure 5:**
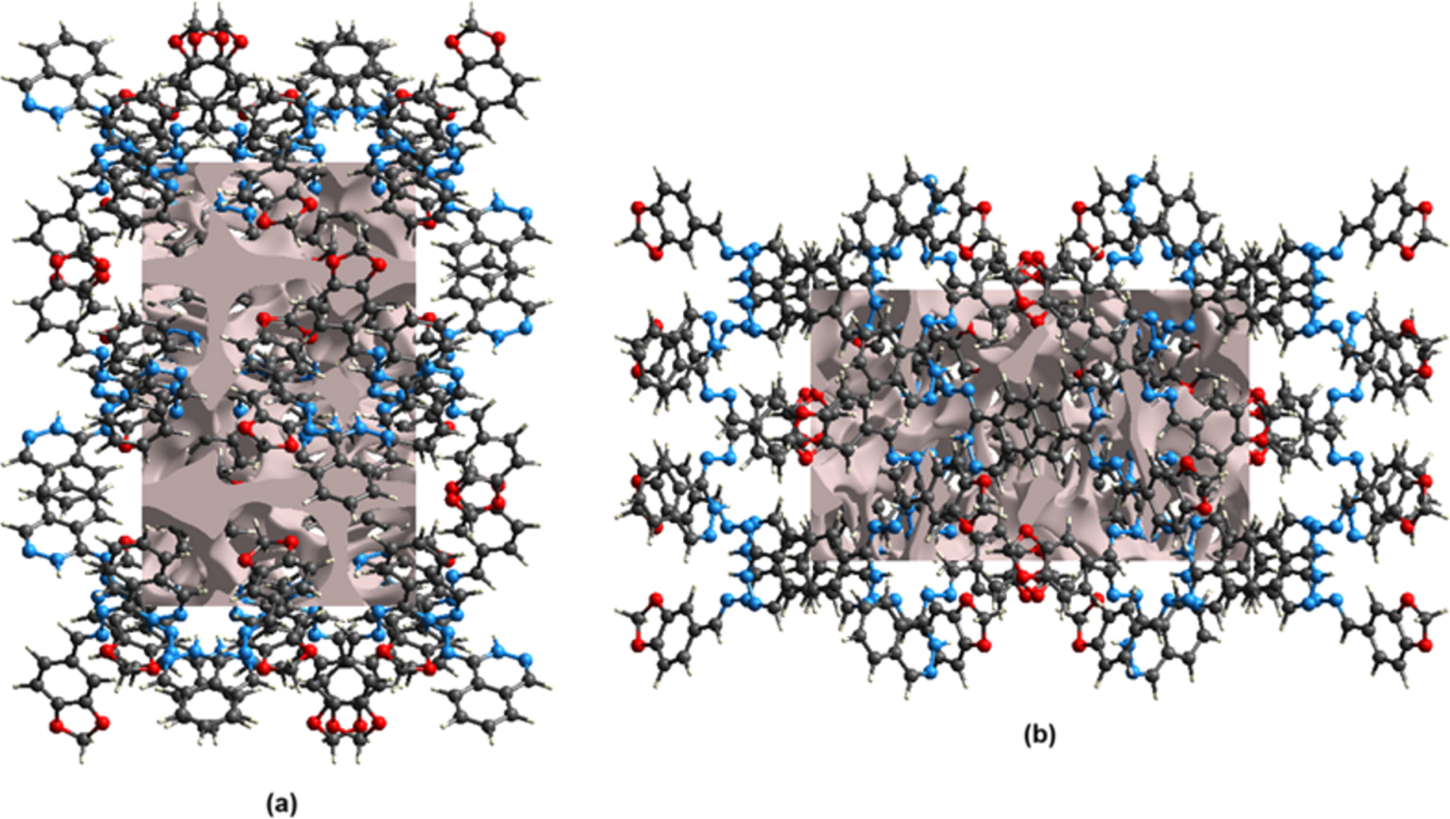
Graphical views of voids in the crystal packing of com­pound view down the (*a*) *a* axis and (*b*) *b* axis.

A void analysis was performed by adding up the electron densities of the spherically symmetric atoms contained in the asymmetric unit (Turner *et al.*, 2011 ▸). The void surface is defined as an isosurface of the procrystal electron density and is calculated for the whole unit cell where the void surface meets the boundary of the unit cell and capping faces are generated to create an enclosed volume. The volumes of the crystal voids (Fig. 5 ▸) and the percentages of free space in the unit cells were calculated to be 614.55 Å^3^ and 11.15%, respectively.

## 5. Synthesis and crystallization

Compound (I) was prepared by condensation of hydralazine hydro­chloride (0.98 g, 0.005 mol) with piperonal (0.75 g, 0.005 mol) in methanol (30 ml). Sodium acetate (0.41 g, 0.005 mol) was added to neutralize the hydralazine hydro­chloride *in situ*, generating free hydralazine; the resulting acetic acid acted as a mild catalyst to promote the reaction. The reaction progress was monitored by thin-layer chromatography (TLC) with ethyl acetate–hexane (5 ml; 1:4 *v*/*v*), con­firming the disappearance of the starting materials. On cooling to room tem­per­a­ture, the crystallized product was collected by vacuum filtration and washed with cold methanol to remove inorganic salts. Single crystals suitable for X-ray diffraction were obtained by recrystallization from di­chloro­methane–methanol (1:1 *v*/*v*) by slow evaporation at room tem­per­a­ture.

## 6. Refinement

Crystal data, data collection and structure refinement details are summarized in Table 2 ▸. All H atoms were placed in idealized positions and allowed to ride on their parent atoms, with N—H = 0.86 Å and C—H = 0.93–0.97 Å, and with *U*_iso_(H) = 1.5*U*_eq_(C) for methyl H atoms and *U*_iso_(H) = 1.2*U*_eq_(C,N) for all other H atoms.

## Supplementary Material

Crystal structure: contains datablock(s) I, shelx. DOI: 10.1107/S2056989026000538/wm5785sup1.cif

Structure factors: contains datablock(s) I. DOI: 10.1107/S2056989026000538/wm5785Isup2.hkl

Supporting information file. DOI: 10.1107/S2056989026000538/wm5785Isup3.cml

CCDC reference: 2512372

Additional supporting information:  crystallographic information; 3D view; checkCIF report

## Figures and Tables

**Table 1:** Hydrogen-bond geometry (Å, °)

*D*—H⋯*A*	*D*—H	H⋯*A*	*D*⋯*A*	*D*—H⋯*A*
N3—H3⋯N1	0.86	2.24	2.596 (2)	105
N3—H3⋯N2^i^	0.86	2.28	3.013 (2)	143
C8—H8⋯N1^ii^	0.93	2.62	3.517 (2)	162

Symmetry codes: (i) 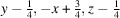; (ii) 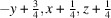.

**Table 2:** Experimental details

Crystal data	
Chemical formula	C_16_H_12_N_4_O_2_
*M*_r_	292.30
Crystal system, space group	Tetragonal, *I*4_1_/*a*
Temperature (K)	300
*a*, *c* (Å)	20.721 (5), 12.835 (3)
*V* (Å^3^)	5511 (3)
*Z*	16
Radiation type	Mo *K*α
μ (mm^−1^)	0.10
Crystal size (mm)	0.33 × 0.13 × 0.12

Data collection	
Diffractometer	Bruker APEXII CCD
Absorption correction	Multi-scan (*SADABS*; Krause *et al.*, 2015 ▸)
*T*_min_, *T*_max_	0.968, 0.988
No. of measured, independent and observed [*I* > 2σ(*I*)] reflections	40086, 3416, 2231
*R*_int_	0.044
(sin θ/λ)_max_ (Å^−1^)	0.666

Refinement	
*R*[*F*^2^ > 2σ(*F*^2^)], *wR*(*F*^2^), *S*	0.048, 0.166, 1.17
No. of reflections	3416
No. of parameters	200
H-atom treatment	H-atom parameters constrained
Δρ_max_, Δρ_min_ (e Å^−3^)	0.18, −0.19

Computer programs: *APEX3* (Bruker, 2017 ▸), *SAINT* (Bruker, 2017 ▸), *SHELXT* (Sheldrick, 2015*a* ▸), *ORTEP-3 for Windows* (Farrugia, 2012 ▸), *SHELXL* (Sheldrick, 2015*b* ▸) and *PLATON* (Spek, 2020 ▸).

## supplementary crystallographic information

### (1Z)-1-[(E)-2-(2H-1,3-Benzodioxol-5-ylmethylidene)hydrazin-1-ylidene]-1,2-dihydrophthalazine Crystal data

**Table:**

C_16_H_12_N_4_O_2_	*D*_x_ = 1.409 Mg m^−^^3^
*M**_r_* = 292.30	Mo *K*α radiation, λ = 0.71073 Å
Tetragonal, *I*4_1_/*a*	Cell parameters from 9881 reflections
*a* = 20.721 (5) Å	θ = 2.7–24.9°
*c* = 12.835 (3) Å	µ = 0.10 mm^−^^1^
*V* = 5511 (3) Å^3^	*T* = 300 K
*Z* = 16	Block, yellow
*F*(000) = 2432	0.33 × 0.13 × 0.12 mm

### (1Z)-1-[(E)-2-(2H-1,3-Benzodioxol-5-ylmethylidene)hydrazin-1-ylidene]-1,2-dihydrophthalazine Data collection

**Table:**

Bruker APEXII CCD diffractometer	2231 reflections with *I* > 2σ(*I*)
Radiation source: i-mu-s microfocus source	*R*_int_ = 0.044
φ and ω scans	θ_max_ = 28.3°, θ_min_ = 1.9°
Absorption correction: multi-scan (SADABS; Krause *et al.*, 2015)	*h* = −27→25
*T*_min_ = 0.968, *T*_max_ = 0.988	*k* = −25→26
40086 measured reflections	*l* = −17→17
3416 independent reflections	

### (1Z)-1-[(E)-2-(2H-1,3-Benzodioxol-5-ylmethylidene)hydrazin-1-ylidene]-1,2-dihydrophthalazine Refinement

**Table:**

Refinement on *F*^2^	Hydrogen site location: inferred from neighbouring sites
Least-squares matrix: full	H-atom parameters constrained
*R*[*F*^2^ > 2σ(*F*^2^)] = 0.048	*w* = 1/[σ^2^(*F*_o_^2^) + (0.0632*P*)^2^ + 3.5875*P*] where *P* = (*F*_o_^2^ + 2*F*_c_^2^)/3
*wR*(*F*^2^) = 0.166	(Δ/σ)_max_ < 0.001
*S* = 1.17	Δρ_max_ = 0.18 e Å^−^^3^
3416 reflections	Δρ_min_ = −0.19 e Å^−^^3^
200 parameters	Extinction correction: SHELXL (Sheldrick, 2015b), Fc^*^=kFc[1+0.001xFc^2^λ^3^/sin(2θ)]^-1/4^
0 restraints	Extinction coefficient: 0.00061 (18)

### (1Z)-1-[(E)-2-(2H-1,3-Benzodioxol-5-ylmethylidene)hydrazin-1-ylidene]-1,2-dihydrophthalazine Special details

**Table:**

Geometry. All esds (except the esd in the dihedral angle between two l.s. planes) are estimated using the full covariance matrix. The cell esds are taken into account individually in the estimation of esds in distances, angles and torsion angles; correlations between esds in cell parameters are only used when they are defined by crystal symmetry. An approximate (isotropic) treatment of cell esds is used for estimating esds involving l.s. planes.

### (1Z)-1-[(E)-2-(2H-1,3-Benzodioxol-5-ylmethylidene)hydrazin-1-ylidene]-1,2-dihydrophthalazine Fractional atomic coordinates and isotropic or equivalent isotropic displacement parameters (Å^2^)

**Table:**

	*x*	*y*	*z*	*U*_iso_*/*U*_eq_	
O1	0.39195 (9)	0.77008 (9)	0.29979 (16)	0.0864 (6)	
O2	0.35150 (8)	0.76292 (8)	0.13220 (14)	0.0724 (5)	
N1	0.18949 (7)	0.56513 (7)	0.13410 (12)	0.0454 (4)	
N2	0.14478 (7)	0.51426 (7)	0.13390 (12)	0.0450 (4)	
N3	0.14684 (8)	0.53064 (8)	−0.04779 (12)	0.0490 (4)	
H3	0.176482	0.559169	−0.038998	0.059*	
N4	0.12757 (9)	0.51996 (9)	−0.14794 (13)	0.0566 (5)	
C1	0.28885 (10)	0.62896 (10)	0.34250 (15)	0.0522 (5)	
H1	0.276323	0.599118	0.392705	0.063*	
C2	0.33250 (11)	0.67694 (11)	0.36977 (17)	0.0609 (6)	
H2	0.349117	0.680115	0.436885	0.073*	
C3	0.34972 (10)	0.71904 (10)	0.29344 (17)	0.0556 (5)	
C4	0.39145 (13)	0.79955 (12)	0.2006 (2)	0.0777 (7)	
H4A	0.435018	0.801540	0.173126	0.093*	
H4B	0.375045	0.843254	0.206206	0.093*	
C5	0.32496 (9)	0.71475 (9)	0.19346 (16)	0.0489 (5)	
C6	0.28210 (9)	0.66826 (9)	0.16482 (15)	0.0466 (4)	
H6	0.266030	0.665787	0.097279	0.056*	
C7	0.26323 (9)	0.62402 (9)	0.24257 (13)	0.0431 (4)	
C8	0.21674 (9)	0.57300 (9)	0.22285 (14)	0.0450 (4)	
H8	0.206329	0.544907	0.276803	0.054*	
C9	0.12438 (8)	0.50119 (9)	0.03975 (14)	0.0419 (4)	
C10	0.08325 (11)	0.47696 (11)	−0.16024 (16)	0.0583 (5)	
H10	0.069081	0.468964	−0.227764	0.070*	
C11	0.05416 (9)	0.44053 (9)	−0.07770 (15)	0.0495 (5)	
C12	0.00633 (11)	0.39398 (11)	−0.09611 (18)	0.0618 (6)	
H12	−0.007580	0.385954	−0.163766	0.074*	
C13	−0.01994 (11)	0.36030 (12)	−0.0145 (2)	0.0653 (6)	
H13	−0.051715	0.329541	−0.026824	0.078*	
C14	0.00088 (11)	0.37211 (11)	0.08663 (19)	0.0613 (6)	
H14	−0.017249	0.349151	0.141587	0.074*	
C15	0.04801 (9)	0.41737 (10)	0.10665 (16)	0.0518 (5)	
H15	0.061712	0.424791	0.174600	0.062*	
C16	0.07506 (8)	0.45203 (9)	0.02410 (14)	0.0436 (4)	

### (1Z)-1-[(E)-2-(2H-1,3-Benzodioxol-5-ylmethylidene)hydrazin-1-ylidene]-1,2-dihydrophthalazine Atomic displacement parameters (Å^2^)

**Table:**

	*U*^11^	*U*^22^	*U*^33^	*U*^12^	*U*^13^	*U*^23^
O1	0.0875 (12)	0.0756 (11)	0.0959 (14)	−0.0334 (9)	−0.0192 (11)	−0.0034 (10)
O2	0.0792 (11)	0.0616 (9)	0.0763 (11)	−0.0167 (8)	0.0059 (9)	0.0119 (8)
N1	0.0493 (9)	0.0468 (8)	0.0401 (8)	−0.0052 (7)	0.0012 (7)	−0.0015 (6)
N2	0.0484 (8)	0.0478 (8)	0.0389 (8)	−0.0049 (7)	−0.0014 (6)	0.0001 (6)
N3	0.0545 (9)	0.0555 (9)	0.0370 (8)	−0.0073 (7)	−0.0045 (7)	0.0026 (7)
N4	0.0643 (11)	0.0672 (11)	0.0384 (9)	−0.0059 (9)	−0.0072 (7)	0.0029 (7)
C1	0.0645 (12)	0.0530 (11)	0.0390 (10)	−0.0027 (9)	−0.0028 (9)	0.0000 (8)
C2	0.0677 (13)	0.0683 (13)	0.0468 (11)	−0.0069 (11)	−0.0130 (10)	−0.0077 (10)
C3	0.0524 (11)	0.0526 (11)	0.0618 (13)	−0.0053 (9)	−0.0043 (9)	−0.0070 (10)
C4	0.0721 (16)	0.0586 (14)	0.102 (2)	−0.0146 (12)	0.0114 (14)	−0.0070 (14)
C5	0.0494 (10)	0.0438 (10)	0.0536 (11)	0.0002 (8)	0.0060 (8)	0.0025 (8)
C6	0.0502 (10)	0.0493 (10)	0.0403 (9)	−0.0006 (8)	−0.0005 (8)	0.0002 (8)
C7	0.0467 (10)	0.0458 (9)	0.0367 (9)	0.0010 (7)	0.0009 (7)	−0.0037 (7)
C8	0.0525 (10)	0.0474 (10)	0.0350 (9)	−0.0031 (8)	0.0020 (7)	−0.0007 (7)
C9	0.0436 (9)	0.0447 (9)	0.0373 (9)	0.0029 (7)	−0.0008 (7)	0.0002 (7)
C10	0.0649 (13)	0.0685 (13)	0.0415 (10)	−0.0071 (11)	−0.0099 (9)	−0.0010 (9)
C11	0.0471 (10)	0.0550 (11)	0.0465 (10)	0.0004 (8)	−0.0040 (8)	−0.0026 (9)
C12	0.0569 (12)	0.0717 (14)	0.0567 (13)	−0.0090 (10)	−0.0100 (10)	−0.0075 (10)
C13	0.0556 (12)	0.0673 (14)	0.0729 (15)	−0.0142 (11)	−0.0042 (11)	−0.0060 (11)
C14	0.0557 (12)	0.0623 (13)	0.0660 (14)	−0.0090 (10)	0.0052 (10)	0.0041 (11)
C15	0.0529 (11)	0.0546 (11)	0.0480 (11)	−0.0040 (9)	−0.0013 (9)	0.0014 (9)
C16	0.0411 (9)	0.0457 (10)	0.0441 (10)	0.0028 (7)	−0.0018 (7)	0.0000 (7)

### (1Z)-1-[(E)-2-(2H-1,3-Benzodioxol-5-ylmethylidene)hydrazin-1-ylidene]-1,2-dihydrophthalazine Geometric parameters (Å, º)

**Table:**

O1—C3	1.375 (3)	C5—C6	1.361 (3)
O1—C4	1.412 (3)	C6—C7	1.410 (3)
O2—C5	1.385 (2)	C6—H6	0.9300
O2—C4	1.425 (3)	C7—C8	1.452 (3)
N1—C8	1.282 (2)	C8—H8	0.9300
N1—N2	1.403 (2)	C9—C16	1.457 (3)
N2—C9	1.308 (2)	C10—C11	1.434 (3)
N3—C9	1.361 (2)	C10—H10	0.9300
N3—N4	1.364 (2)	C11—C16	1.397 (3)
N3—H3	0.8600	C11—C12	1.403 (3)
N4—C10	1.289 (3)	C12—C13	1.371 (3)
C1—C2	1.389 (3)	C12—H12	0.9300
C1—C7	1.392 (3)	C13—C14	1.389 (3)
C1—H1	0.9300	C13—H13	0.9300
C2—C3	1.359 (3)	C14—C15	1.378 (3)
C2—H2	0.9300	C14—H14	0.9300
C3—C5	1.385 (3)	C15—C16	1.397 (3)
C4—H4A	0.9700	C15—H15	0.9300
C4—H4B	0.9700		
			
C3—O1—C4	105.94 (18)	C1—C7—C8	117.84 (17)
C5—O2—C4	105.36 (18)	C6—C7—C8	122.25 (16)
C8—N1—N2	112.83 (15)	N1—C8—C7	122.66 (17)
C9—N2—N1	111.74 (15)	N1—C8—H8	118.7
C9—N3—N4	127.26 (16)	C7—C8—H8	118.7
C9—N3—H3	116.4	N2—C9—N3	124.01 (17)
N4—N3—H3	116.4	N2—C9—C16	119.91 (16)
C10—N4—N3	115.84 (17)	N3—C9—C16	116.08 (16)
C2—C1—C7	122.22 (19)	N4—C10—C11	124.95 (18)
C2—C1—H1	118.9	N4—C10—H10	117.5
C7—C1—H1	118.9	C11—C10—H10	117.5
C3—C2—C1	116.66 (19)	C16—C11—C12	119.59 (19)
C3—C2—H2	121.7	C16—C11—C10	118.10 (18)
C1—C2—H2	121.7	C12—C11—C10	122.31 (19)
C2—C3—O1	128.2 (2)	C13—C12—C11	120.1 (2)
C2—C3—C5	121.96 (19)	C13—C12—H12	119.9
O1—C3—C5	109.88 (19)	C11—C12—H12	119.9
O1—C4—O2	109.22 (19)	C12—C13—C14	120.0 (2)
O1—C4—H4A	109.8	C12—C13—H13	120.0
O2—C4—H4A	109.8	C14—C13—H13	120.0
O1—C4—H4B	109.8	C15—C14—C13	120.9 (2)
O2—C4—H4B	109.8	C15—C14—H14	119.5
H4A—C4—H4B	108.3	C13—C14—H14	119.5
C6—C5—O2	128.04 (19)	C14—C15—C16	119.52 (19)
C6—C5—C3	122.52 (18)	C14—C15—H15	120.2
O2—C5—C3	109.43 (18)	C16—C15—H15	120.2
C5—C6—C7	116.73 (17)	C11—C16—C15	119.81 (17)
C5—C6—H6	121.6	C11—C16—C9	117.76 (17)
C7—C6—H6	121.6	C15—C16—C9	122.43 (17)
C1—C7—C6	119.91 (17)		
			
C8—N1—N2—C9	−171.01 (16)	C6—C7—C8—N1	−0.6 (3)
C9—N3—N4—C10	−0.2 (3)	N1—N2—C9—N3	3.8 (3)
C7—C1—C2—C3	−0.5 (3)	N1—N2—C9—C16	−176.73 (14)
C1—C2—C3—O1	−179.5 (2)	N4—N3—C9—N2	−179.39 (18)
C1—C2—C3—C5	0.3 (3)	N4—N3—C9—C16	1.1 (3)
C4—O1—C3—C2	−178.2 (2)	N3—N4—C10—C11	−0.4 (3)
C4—O1—C3—C5	1.9 (3)	N4—C10—C11—C16	−0.1 (3)
C3—O1—C4—O2	−3.8 (3)	N4—C10—C11—C12	−179.6 (2)
C5—O2—C4—O1	4.2 (3)	C16—C11—C12—C13	0.4 (3)
C4—O2—C5—C6	178.1 (2)	C10—C11—C12—C13	179.9 (2)
C4—O2—C5—C3	−3.0 (2)	C11—C12—C13—C14	−0.2 (4)
C2—C3—C5—C6	−0.2 (3)	C12—C13—C14—C15	−0.2 (4)
O1—C3—C5—C6	179.64 (18)	C13—C14—C15—C16	0.2 (3)
C2—C3—C5—O2	−179.1 (2)	C12—C11—C16—C15	−0.3 (3)
O1—C3—C5—O2	0.7 (2)	C10—C11—C16—C15	−179.82 (18)
O2—C5—C6—C7	179.09 (18)	C12—C11—C16—C9	−179.46 (18)
C3—C5—C6—C7	0.4 (3)	C10—C11—C16—C9	1.0 (3)
C2—C1—C7—C6	0.7 (3)	C14—C15—C16—C11	0.0 (3)
C2—C1—C7—C8	−178.53 (19)	C14—C15—C16—C9	179.12 (18)
C5—C6—C7—C1	−0.6 (3)	N2—C9—C16—C11	179.03 (17)
C5—C6—C7—C8	178.62 (17)	N3—C9—C16—C11	−1.5 (2)
N2—N1—C8—C7	−178.01 (16)	N2—C9—C16—C15	−0.1 (3)
C1—C7—C8—N1	178.68 (18)	N3—C9—C16—C15	179.37 (17)

### (1Z)-1-[(E)-2-(2H-1,3-Benzodioxol-5-ylmethylidene)hydrazin-1-ylidene]-1,2-dihydrophthalazine Hydrogen-bond geometry (Å, º)

**Table:**

*D*—H···*A*	*D*—H	H···*A*	*D*···*A*	*D*—H···*A*
N3—H3···N1	0.86	2.24	2.596 (2)	105
N3—H3···N2^i^	0.86	2.28	3.013 (2)	143
C8—H8···N1^ii^	0.93	2.62	3.517 (2)	162

Symmetry codes: (i) *y*−1/4, −*x*+3/4, *z*−1/4; (ii) −*y*+3/4, *x*+1/4, *z*+1/4.
